# Dynamic Relationship Between Sleep Patterns and Behavioral and Psychological Symptoms of Dementia: Longitudinal Observational Study

**DOI:** 10.2196/80422

**Published:** 2026-03-23

**Authors:** Eunhee Cho, Sinwoo Hwang, Minhee Yang, Eunkyo Kim, Jungwon Cho, Chang Park

**Affiliations:** 1 Mo-Im Kim Nursing Research Institute College of Nursing Yonsei University Seoul Republic of Korea; 2 Army Cadet Military School Goesan Republic of Korea; 3 College of Nursing and Brain Korea 21 FOUR Project Yonsei University Seoul Republic of Korea; 4 College of Nursing University of Illinois Chicago Chicago, IL United States

**Keywords:** behavioral and psychological symptoms, dementia, granger causality, panel vector autoregressive model, sleep

## Abstract

**Background:**

A higher prevalence of behavioral and psychological symptoms of dementia is associated with a greater caregiver burden and increased mortality in people with dementia. Considering the possibility of a reciprocal relationship between sleep disturbances and these symptoms, time series analyses are necessary to explore the associated temporal dynamics.

**Objective:**

This study aimed to examine dynamic interdependencies between sleep disturbances and behavioral and psychological symptoms of dementia in older adults.

**Methods:**

Daily interactions between sleep patterns and behavioral and psychological symptoms of dementia were analyzed over a 14-day period using a panel vector autoregressive model. Data were collected from June 2018 to June 2020 in community and institutional settings. A total of 154 older adults with dementia wore wrist actigraphy devices continuously for 2 weeks for sleep data, and caregivers recorded behavioral and psychological symptoms of dementia in a daily symptom diary.

**Results:**

Using a panel vector autoregressive model, we analyzed data from 154 older adults living with dementia and their caregivers. The results showed unidirectional Granger causality running from the number of awakenings on the previous day to irritability (*P*=.03) and appetite or eating disorders (*P*=.04) on the following day. Conversely, some of the previous day’s behavioral and psychological symptoms of dementia temporally preceded subsequent changes in sleep patterns. Specifically, delusions had a Granger-causality effect on total sleep time (*P*<.001), wake after sleep onset (*P*=.01), and the number of awakenings (*P*=.006), while irritability had a Granger causality effect on the number of awakenings (*P*=.007). Notably, bidirectional Granger causality was observed between irritability and the number of awakenings.

**Conclusions:**

This study demonstrates that the relationship between the behavioral and psychological symptoms of dementia and sleep patterns is dynamic and forms a vicious cycle. Consequently, early intervention to alleviate symptoms is imperative, and strategies to enhance sleep quality and address sleep disturbances should be prioritized.

## Introduction

Behavioral and psychological symptoms of dementia encompass a range of noncognitive disturbances [1]. The Neuropsychiatric Inventory, a scale widely used for assessing the behavioral and psychological symptoms of dementia, identifies 12 subsymptoms, including delusion, hallucination, anxiety, irritability, depression, sleep disturbances, apathy, disinhibition, changes in appetite, and agitation [1,2]. These behavioral and psychological symptoms can manifest at any stage of dementia, with their prevalence, frequency, and severity often increasing as the condition progresses [3]. Recent evidence indicates that approximately 90% of people living with dementia exhibit at least 1 behavioral and psychological symptom [4]. A higher prevalence of such symptoms is associated with a higher care burden in caregivers and increased mortality among people living with dementia [5-7]. In light of these observations, securing effective management of the behavioral and psychological symptoms of dementia is crucial to alleviating caregiver burden and improving the quality of life for those living with dementia. Among the factors contributing to behavioral and psychological symptoms of dementia, unmet physical and psychological needs have been identified as key drivers [8,9], with sleep disturbances playing a particularly important role. Individuals living with dementia commonly experience sleep disturbances [10,11], and accumulating evidence indicates that poor sleep quality is associated with behavioral and psychological symptoms of dementia, such as apathy and depression [12-14]. The existing evidence suggests that sleep patterns may correlate with the occurrence of behavioral and psychological symptoms of dementia [15-17].

Several studies have explored the relationship between sleep disturbances and the behavioral and psychological symptoms of dementia. Some studies suggest that sleep disturbances contribute to the development of such symptoms [18-20], while others claim that such symptoms may affect sleep disturbances the following day [12]. Cho et al [20] identified that sleep disturbances are a risk factor for behavioral and psychological symptoms of dementia in patients living with the condition. Nonetheless, these results have definite drawbacks because they assume a unidirectional relationship. Given the complexity of these interactions, there is a need to examine the temporal dynamics and directionality of the relationship between sleep patterns and behavioral and psychological symptoms of dementia.

Accordingly, this study used a panel vector autoregressive approach to examine day-to-day temporal dynamics between actigraphy-based sleep patterns and caregiver-recorded behavioral and psychological subsymptoms of dementia, using data collected over a 14-day period. Given the potential variability in the associations between behavioral and psychological subsymptoms of dementia and sleep patterns, we initially used a linear mixed model to account for between-participant variability. However, this approach is limited in its ability to capture dynamic changes over time and directional temporal associations. To address these limitations, we subsequently used a panel vector autoregressive model, which treats all variables as endogenous and enables the examination of reciprocal temporal interdependencies and autoregressive structures over time. This approach is well suited for exploratory analyses of complex temporal relationships without imposing predefined causal directions [21]. Accordingly, this approach allowed for an exploratory examination of day-to-day temporal dynamics between objectively measured sleep disturbances and behavioral and psychological symptoms of dementia, including 12 specific subsymptoms. By applying this framework, this study aims to provide a more nuanced understanding of the temporal interdependence between behavioral and psychological symptoms of dementia and sleep disturbances at the daily level.

## Methods

### Design

This study used a prospective observational design and applied a panel vector autoregressive model to explore the dynamic interdependencies between behavioral and psychological symptoms of dementia and sleep patterns.

### Participants

Older adults with dementia were recruited from neurology outpatient departments of tertiary hospitals, daycare centers, and nursing homes in South Korea. The inclusion criteria for the participants were as follows: age 65 years or older, a diagnosis of dementia, a Korean Mini-Mental State Examination score less than 24 points, and the presence of behavioral and psychological symptoms of dementia at least once a week at baseline. Primary caregivers were also included if they provided the majority of care for the recruited older adults and were able to read and write in Korean. The data were collected from June 2018 to June 2020.

### Procedure

Eligibility screening was conducted by trained researchers through medical record reviews and participant interviews. Two researchers visited the participants’ homes or nursing homes on 2 occasions to collect data. Demographic and health data were collected during the first visit. Moreover, older adults living with dementia were instructed to wear an actigraphy device on their wrists continuously for 2 weeks, and caregivers were asked to record the behavioral and psychological symptoms of dementia in a daily diary for 2 weeks. The researchers conducted telephone follow-ups with participants and their caregivers at least 3 times during the 2-week period to monitor participation status and document any notable observations or issues. At the second visit, which was conducted 2 weeks later, the behavioral and psychological symptoms of dementia were retrieved from the diaries and actigraphy devices for data extraction and analysis.

### Measurements

#### Demographic and Health Data

Demographic data, including age, sex, marital status, and education level, along with information on activities of daily living, Mini-Mental State Examination scores, use of sedative medication, and type of dementia diagnosis, were collected from the caregivers and participants’ medical records.

#### Behavioral and Psychological Symptoms of Dementia

A diary consisting of checklists was developed based on the Neuropsychiatric Inventory [22], a standardized instrument for measuring 12 neuropsychiatric symptoms in people living with dementia. Caregivers were asked to record behavioral and psychological symptoms of dementia in a diary daily for 2 weeks. These symptoms were delusions, hallucinations, agitation or aggression, depression, anxiety, euphoria or elation, apathy, disinhibition, irritability, aberrant motor behavior, sleep and nighttime behavior, and appetite or eating disorders.

#### Sleep Patterns

Sleep patterns were objectively measured using an actigraphy device, the wGT3X-BT activity monitor (ActiGraph) [23]. Participants wore the device on their wrists for 2 weeks, and sleep data were extracted by applying the Cole-Kripke algorithm in ActiLife (version 6.13.3; Ametris) software. The following variables were derived from the daily sleep pattern data, with midnight as the reference point: total sleep time (ie, the time measured as sleep in a day), wake after sleep onset (ie, the time measured as wakefulness during sleep), the number of awakenings, and mean awakening length (ie, the average length of each awakening in minutes).

### Ethical Considerations

The data collection procedures for the original study received ethics approval from the institutional review boards of Severance Hospital (4-2018-0348, 4-2019-0314, and 4-2020-0454) and Ilsan Hospital (2018-10-002-001 and 2019-08-012-001). In the original study, informed consent procedures included explicit permission for future secondary analyses of the collected data without the need for additional consent. Participants who completed the study received approximately US $38 as compensation for their participation. All data used in this study were deidentified before access by the research team. Study-related documents and electronic files were stored on a secure, password-protected computer with access limited to members of the authorized research team.

### Statistical Analyses

All statistical analyses were performed using Stata (version 16.1; Stata Corp). Given the substantial individual variability in the behavioral and psychological symptoms of dementia and sleep patterns, we first applied a linear mixed model to account for between-participant differences and examine individual-level heterogeneity. We calculated the intraclass correlation coefficient (ICC) to quantify the proportion of the total variance attributable to individual differences. This coefficient indicates the extent to which the variability in a construct is explained by between-participant differences, rather than within-individual fluctuations. A higher ICC suggests that individual characteristics contribute to the observed variability in the behavioral and psychological symptoms of dementia and sleep patterns. However, as the linear mixed model has limitations in capturing bidirectional relationships, fully reflecting dynamic interactions over time, and testing causality, we further used the panel vector autoregressive model to analyze the dynamic interplay between behavioral and psychological symptoms of dementia and sleep patterns.

For the panel vector autoregressive estimation, the optimal lag length was determined as lag 1 based on the minimum modified Bayesian information criterion, modified Akaike information criterion, and modified Hannan-Quinn information criterion statistics [24] (Table S1 in Multimedia Appendix 1). To assess causality within this framework, we conducted a Granger causality test, where the null hypothesis assumed no causal relationship, and a *P* value <.05 indicated rejection of the null hypothesis (ie, there was a Granger causal effect). This test also allowed us to determine whether the relationship was unidirectional or bidirectional. Statistical significance was set at *P*<.05.

## Results

### Participants’ General Characteristics, Behavioral and Psychological Symptoms of Dementia, and Sleep Patterns

We initially recruited 175 older adults with dementia, but 18 (10.3%) were excluded owing to refusal to wear the actigraphy device (n=10, 55.6%), hospitalization (n=6, 33.3%), death (n=1, 5.6%), and absence of a caregiver during data collection (n=1, 5.6%). We also excluded 3 (1.7%) participants with insufficient data from the actigraphy device, leaving a final sample of 154 (88%) older adults living with dementia and their caregivers.

Table S2 in Multimedia Appendix 1 shows the baseline characteristics of the participants. The mean age was 81.34 (SD 5.97) years, 93 (60.4%) were women, and most had an elementary school education or less (n=70, 49.4%). The mean activities of daily living score was 10.68 (SD 3.87), indicating difficulty in performing activities of daily living. The mean Mini-Mental State Examination score was 16.86 (SD 5.84), indicating moderate cognitive impairment. Regarding the type of dementia diagnosis, 76 (49.4%) participants were diagnosed with Alzheimer disease, 60 (39%) with Lewy body dementia, 24 (15.6%) with vascular dementia, and 34 (22.1%) with other types of dementia.

Table 1 shows the participants’ behavioral and psychological symptoms of dementia and sleep patterns, with a mean of 12.85 (SD 2.40) days of data recorded per participant. Of the 2145 days on which symptoms were recorded, apathy was the most common symptom (n=293, 13.7%), followed by sleep and nighttime behavior (n=259, 12.1%), anxiety (n=238, 11.1%), irritability (n=231, 10.8%), depression (n=230, 10.7%), agitation or aggression (n=194, 9%), and delusion (n=192, 8.6%). The mean total sleep time was 458.21 (SD 253.05) minutes, with a mean of 29.37 (SD 27.47) minutes spent awake during sleep. The total number of awakenings was 12.40 (SD 10.25) per night, with each awakening lasting approximately 2.16 (SD 1.09) minutes.

**Table 1:** Participants’ behavioral and psychological symptoms of dementia and sleep patterns (N=2145 days).

Variables		Values
Number of days of behavioral and psychological symptoms of dementia and actigraphy recordings per person, mean (SD)		12.85 (2.40)
**Behavioral and psychological symptoms of dementia, n (%)**		
	Delusion	192 (8.6)
	Hallucination	129 (5.9)
	Agitation or aggression	194 (9.0)
	Depression	230 (10.7)
	Anxiety	238 (11.1)
	Euphoria or elation	108 (4.9)
	Apathy	293 (13.7)
	Disinhibition	41 (1.9)
	Irritability	231 (10.8)
	Aberrant motor behavior	73 (3.4)
	Sleep and nighttime behavior	259 (12.1)
	Appetite or eating disorders	157 (7.3)
**Sleep factors, mean (SD)**		
	Total sleep time (minutes)	458.21 (253.05)
	Wake after sleep onset (minutes)	29.37 (27.47)
	Number of awakenings	12.40 (10.25)
	Mean awake length (minutes)	2.16 (1.09)

### Linear Mixed Model Estimation Results for Total Behavioral and Psychological Symptoms of Dementia and Sleep Patterns

On the basis of the linear mixed model analysis (Table S3 in Multimedia Appendix 1), there was substantial between-participant variability in the behavioral and psychological symptoms of dementia; however, the fixed effects of sleep patterns on these symptoms were not statistically significant in this model. The ICC values suggested that approximately 49% to 50% of the total variance in the behavioral and psychological symptoms of dementia can be attributed to between-participant differences rather than within-participant fluctuations over time. The random slopes were statistically significant, indicating that the effect of the previous day’s symptoms on the following day’s symptoms varied between individuals.

Similarly, the total behavioral and psychological symptoms of dementia did not have a statistically significant fixed effect on sleep patterns (Table S4 in Multimedia Appendix 1). However, the ICCs ranged from 0.097 to 0.391, indicating that individual-level differences contributed moderately to the overall variance in sleep patterns, particularly for wake after sleep onset (0.339) and the number of awakenings (0.391), where the proportion of variance due to individual differences was relatively high.

Given the statistically significant random slopes, the relationship between the behavioral and psychological symptoms of dementia and sleep may be strongly influenced by individual differences. This required further analysis using a panel vector autoregressive model to capture the within-individual temporal dynamics.

### Panel Vector Autoregressive Model Estimation Results for Total Behavioral and Psychological Symptoms of Dementia and Sleep Patterns

Table 2 shows the results of the panel vector autoregressive model estimation, with behavioral and psychological symptoms of dementia as the response variable. The previous day’s wake after sleep onset (*P*=.01), the number of awakenings (*P*=.008), and mean awakening length (*P*=.008) were temporally associated with higher levels of the following day’s total behavioral and psychological symptoms of dementia.

**Table 2:** Panel vector autoregressive model estimation results for total behavioral and psychological symptoms of dementia by sleep pattern.

Impulse variables	Response variables: total behavioral and psychological symptoms of dementia	
	β (SE)	*P* value^a^
Total sleep time	0.00089 (0.00056)	.11
Wake after sleep onset	0.00495 (0.00200)	.01
Number of awakenings	0.01580 (0.00597)	.008
Mean awakening length	0.15242 (0.05767)	.008

^a^*P* value represent the results of the Granger causality test within the panel vector autoregressive model.

Table 3 shows the results of the panel vector autoregressive model estimation using sleep patterns as the response variable. The previous day’s behavioral and psychological symptoms of dementia were temporally associated with higher total sleep time (*P*<.001), greater wake after sleep onset (*P*=.003), and a higher number of awakenings (*P*=.001) on the following day.

**Table 3:** Panel vector autoregressive model estimation results for sleep pattern by total behavioral and psychological symptoms of dementia.

Impulse variables: total behavioral and psychological symptoms of dementia	Response variables	
	β (SE)	*P* value^a^
Total sleep time	90.49515 (25.21297)	<.001
Wake after sleep onset	4.34126 (1.47666)	.003
Number of awakenings	1.83004 (0.56304)	.001
Mean awakening length	0.00914 (0.07713)	.91

^a^*P* value represent the results of the Granger causality test within the panel vector autoregressive model.

Granger causality tests (Tables 2 and 3) were conducted to test for causality between the variables that had a statistically significant relationship. The tests revealed unidirectional Granger causality, consistent with the panel vector autoregressive model estimates.

### Linear Mixed Model Estimation Results for Behavioral and Psychological Subsymptoms of Dementia and Sleep Patterns

Tables S5 and S6 in Multimedia Appendix 1 show the relationship between the 12 behavioral and psychological subsymptoms of dementia and sleep patterns as analyzed by the linear mixed model. The findings resemble those of the linear mixed model analysis of total symptoms and sleep patterns. Few of the fixed effects of sleep patterns on the behavioral and psychological subsymptoms of dementia were statistically significant, but the ICC values ranged from 0.57 to 0.83, indicating that much of the variation in the subsymptoms could be attributed to between-individual differences (Table S5 in Multimedia Appendix 1).

Similarly, the 12 behavioral and psychological subsymptoms of dementia did not exhibit a statistically significant fixed effect on sleep patterns (Table S6 in Multimedia Appendix 1). However, the ICC values, ranging from 0.099 to 0.391, suggest that individual differences account for a moderate proportion of the overall variance in sleep patterns. Notably, wake after sleep onset (ICC 0.337-0.340) and the number of awakenings (ICC 0.389-0.391) showed relatively high ICC values, indicating that a substantial portion of the variance in these sleep parameters is attributable to between-participant variability.

The high ICC values and statistically significant random slopes indicate substantial individual variability in the relationships between behavioral and psychological subsymptoms of dementia and sleep patterns. Consequently, it was necessary to use a panel vector autoregressive model to analyze these within-individual dynamic, reciprocal relationships over time.

### Panel Vector Autoregressive Model Estimation Results for Behavioral and Psychological Subsymptoms of Dementia and Sleep Patterns

Tables 4-6 show the results of the panel vector autoregressive model estimation for behavioral and psychological subsymptoms of dementia as response variables. The previous day’s sleep patterns were temporally associated with higher levels of some subsymptoms: irritability was temporally associated with higher total sleep time (*P*=.02), a greater number of awakenings (*P*=.03), and longer mean awakening length (*P*=.04) on the previous day, and appetite or eating disorders were temporally associated with a higher number of awakenings on the previous day (*P*=.04).

**Table 4:** Panel vector autoregressive model estimation results for the behavioral and psychological subsymptoms (delusion, hallucination, agitation or aggression, and depression) of dementia by sleep pattern.

Response and impulse variables		β (SE)	*P* value^a^
**Delusion**			
	TST^b^	0.00026 (0.00016)	.10
	WASO^c^	0.00090 (0.00058)	.12
	NoA^d^	0.00249 (0.00167)	.13
	MAL^e^	0.01977 (0.01654)	.23
**Hallucination**			
	TST	0.00004 (0.00013)	.78
	WASO	0.00048 (0.00058)	.40
	NoA	0.00197 (0.00178)	.27
	MAL	0.01163 (0.01269)	.36
**Agitation or aggression**			
	TST	0.00003 (0.00016)	.87
	WASO	0.00018 (0.00055)	.75
	NoA	0.00061 (0.00177)	.73
	MAL	–0.00100 (0.01699)	.95
**Depression**			
	TST	–0.00016 (0.00018)	.37
	WASO	–0.00033 (0.00070)	.64
	NoA	–0.00113 (0.00208)	.59
	MAL	0.00441 (0.01972)	.82

^a^*P* value represent the results of the Granger causality test within the panel vector autoregressive model.

^b^TST: total sleep time.

^c^WASO: wake after sleep onset.

^d^NoA: number of awakenings.

^e^MAL: mean awakening length.

**Table 5:** Panel vector autoregressive model estimation results for the behavioral and psychological subsymptoms (anxiety, euphoria or elation, apathy, and disinhibition) of dementia by sleep pattern.

Response and impulse variables			β (SE)		*P* value^a^		
**Anxiety**							
	TST^b^	–0.00018 (0.00018)		.33			
	WASO^c^	0.00015 (0.00057)		.79			
	NoA^d^	0.00022 (0.00162)		.89			
	MAL^e^	0.01034 (0.01991)		.60			
**Euphoria or elation**							
	TST	0.00014 (0.00013)		.28			
	WASO	0.00059 (0.00047)		.20			
	NoA	0.00207 (0.00138)		.13			
	MAL	0.02514 (0.01582)		.11			
**Apathy**							
	TST	0.00020 (0.00018)		.25			
	WASO	0.00058 (0.00074)		.43			
	NoA	0.00288 (0.00221)		.19			
	MAL	0.00412 (0.01818)		.82			
**Disinhibition**							
	TST	–0.00013 (0.00009)		.15			
	WASO	–0.00038 (0.00040)		.34			
	NoA	0.87058 (2.85687)		.76			
	MAL	–0.00547 (0.00925)		.55			

^a^*P* value represent the results of the Granger causality test within the panel vector autoregressive model.

^b^TST: total sleep time.

^c^WASO: wake after sleep onset.

^d^NoA: number of awakenings.

^e^MAL: mean awakening length.

**Table 6:** Panel vector autoregressive model estimation results for the behavioral and psychological subsymptoms of dementia (irritability, aberrant motor behavior, sleep and nighttime behavior, and appetite or eating disorders) by sleep pattern.

Response and impulse variables		β (SE)	*P* value^a^
**Irritability**			
	TST^b^	0.00048 (0.00021)	.02
	WASO^c^	0.00126 (0.00065)	.05
	NoA^d^	0.00428 (0.00199)	.03
	MAL^e^	0.03738 (0.01821)	.04
**Aberrant motor behavior**			
	TST	0.00004 (0.00009)	.61
	WASO	0.00001 (0.00038)	.97
	NoA	0.00112 (0.0011232)	.94
	MAL	0.00049 (0.01183)	.97
**Sleep and nighttime behavior**			
	TST	0.00008 (0.00018)	.66
	WASO	0.00130 (0.00078)	.09
	NoA	0.00347 (0.00222)	.12
	MAL	0.03416 (0.01947)	.08
**Appetite or eating disorders**			
	TST	0.00020 (0.00013)	.13
	WASO	0.00091 (0.00048)	.06
	NoA	0.00292 (0.00141)	.04
	MAL	0.02892 (0.01586)	.07

^a^*P* value represent the results of the Granger causality test within the panel vector autoregressive model.

^b^TST: total sleep time.

^c^WASO: wake after sleep onset.

^d^NoA: number of awakenings.

^e^MAL: mean awakening length.

Table 7 shows the results of the panel vector autoregressive model estimation for sleep patterns as the response variable. Some of the previous day’s behavioral and psychological subsymptoms of dementia were temporally associated with changes in sleep patterns on the following day: the previous day’s delusion (*P*<.001), hallucination (*P*=.04), agitation or aggression (*P*=.02), depression (*P*=.002), anxiety (*P*=.02), and apathy (*P*=.04) were temporally associated with higher total sleep time; the previous day’s delusion (*P*=.01), agitation or aggression (*P*=.02), and apathy (*P*=.02) were temporally associated with greater wake after sleep onset; and the previous day’s delusions (*P*=.006), apathy (*P*=.02), and irritability (*P*=.007) were temporally associated with a higher number of awakenings on the following day.

**Table 7:** Panel vector autoregressive model estimation results for sleep pattern by the behavioral and psychological subsymptoms of dementia.

Response and impulse variables		β (SE)	*P* value^a^
**Total sleep time**			
	Delusion	207.12760 (59.37694)	<.001
	Hallucination	146.51250 (69.72166)	.04
	Agitation or aggression	148.64660 (61.71230)	.02
	Depression	186.97850 (59.76790)	.002
	Anxiety	166.45380 (71.14920)	.02
	Euphoria or elation	59.388490 (59.88787)	.32
	Apathy	128.96060 (61.87693)	.04
	Disinhibition	–44.88155 (149.0512)	.76
	Irritability	82.21647 (53.16712)	.12
	Aberrant motor behavior	141.56140 (82.73073)	.09
	Sleep and nighttime behavior	113.56550 (59.04195)	.05
	Appetite or eating disorders	11.843420 (60.79683)	.85
**Wake after sleep onset**			
	Delusion	8.55337 (3.44196)	.01
	Hallucination	7.06856 (4.91151)	.15
	Agitation or aggression	9.17265 (4.03159)	.02
	Depression	1.671489 (3.45359)	.63
	Anxiety	–1.32085 (4.10093)	.75
	Euphoria or elation	3.72705 (4.00864)	.35
	Apathy	10.78888 (4.53788)	.02
	Disinhibition	5.65713 (8.89465)	.80
	Irritability	5.657127 (3.06763)	.07
	Aberrant motor behavior	7.76621 (6.07391)	.20
	Sleep and nighttime behavior	6.09917 (3.51403)	.08
	Appetite or eating disorders	5.15131 (3.93965)	.19
**Number of awakenings**			
	Delusion	3.79514 (1.39367)	.006
	Hallucination	2.83285 (1.48000)	.14
	Agitation or aggression	2.75541 (1.43263)	.05
	Depression	0.90918 (1.30923)	.49
	Anxiety	–0.60624 (1.52338)	.69
	Euphoria or elation	1.73797 (1.53151)	.26
	Apathy	4.07411 (1.67891)	.02
	Disinhibition	0.87058 (2.85687)	.76
	Irritability	3.31969 (1.23878)	.007
	Aberrant motor behavior	3.79111 (2.51553)	.13
	Sleep and nighttime behavior	2.28761 (1.32894)	.09
	Appetite or eating disorders	2.23741 (1.51776)	.14
**Mean awakening length**			
	Delusion	0.20611 (0.18999)	.28
	Hallucination	–0.08000 (0.22148)	.72
	Agitation or aggression	0.12385 (0.21356)	.56
	Depression	0.09569 (0.15077)	.53
	Anxiety	0.04655 (0.24929)	.85
	Euphoria or elation	–0.02885 (0.19814)	.88
	Apathy	0.13391 (0.18566)	.47
	Disinhibition	–0.25250 (0.45123)	.58
	Irritability	–0.12203 (0.16155)	.45
	Aberrant motor behavior	0.03143 (0.29223)	.91
	Sleep and nighttime behavior	–0.04485 (0.20128)	.82
	Appetite or eating disorders	–0.21659 (0.20477)	.29

^a^*P* value represent the results of the Granger causality test within the panel vector autoregressive model.

Granger causality tests were subsequently conducted for variable pairs that demonstrated statistically significant temporal associations in the panel vector autoregressive models (Tables 5-7). The results showed unidirectional Granger causality, consistent with the panel vector autoregressive model estimation. Bidirectional Granger causality was observed only between irritability and the number of awakenings.

## Discussion

### Principal Findings

Using panel data and a panel vector autoregressive model, this study longitudinally analyzed the dynamic relationships between behavioral and psychological symptoms of dementia and sleep patterns among older adults with dementia. Regarding the main findings, first, the number of sleep awakenings on the previous day was temporally associated with higher levels of irritability and appetite or eating disorders on the following day. Second, some of the previous day’s behavioral and psychological subsymptoms of dementia were temporally associated with subsequent changes in sleep patterns (eg, an increase in total sleep time, the number of awakenings, and mean awakening length).

A linear mixed model analysis was conducted to contextualize these findings, revealing substantial between-participant variability in both behavioral and psychological symptoms of dementia and sleep patterns. Although the fixed effects were not statistically significant, the random slopes were statistically significant, and there were moderate to high ICCs, indicating that individual differences contributed meaningfully to variability in both variables; that is, the relationships between behavioral and psychological symptoms of dementia and sleep patterns were not uniform across individuals and may involve complex, time-dependent interactions. Accordingly, a panel vector autoregressive model was implemented, revealing statistically significant temporal associations in which sleep patterns preceded changes in irritability and appetite or eating disorders. This indicates that interventions to improve sleep quality should be implemented in the early stages of sleep disturbance to alleviate the behavioral and psychological symptoms of dementia that may stem from poor sleep quality. Importantly, our evidence implies that effectively preventing the occurrence of irritability and appetite or eating disorders in older adults with dementia requires careful and consistent monitoring and management of increases in total sleep time, the number of awakenings, and mean awakening length. Previous studies have reported that 22.6% of people with dementia have excessive daytime sleepiness and that sleep disturbances are associated with the onset of behavioral and psychological symptoms of dementia [18,19]. Furthermore, to avoid excessive total sleep time, people with dementia should engage in sufficient physical and social activity and limit daytime napping [25,26].

In particular, the number of awakenings during sleep and their duration demonstrated significant temporal associations with subsequent behavioral and psychological symptoms. These findings support other studies that have found a relationship between excessive awakenings and the occurrence of behavioral and psychological symptoms of dementia [27]. Education on sleep hygiene practices, such as maintaining consistent sleep-wake routines and optimizing the sleep environment [25], and combined nonpharmacological interventions, including physical or social activities and caregiver support interventions [28], are reportedly effective in reducing awakenings during sleep and alleviating the behavioral and psychological symptoms of dementia.

Conversely, behavioral and psychological symptoms of dementia onset were temporally associated with subsequent sleep patterns. In particular, irritability had bidirectional Granger causality with the number of awakenings, implying that poor sleep patterns may contribute to the occurrence of symptoms the next day and that the occurrence of irritability may increase the number of awakenings, their duration, and excessive total sleep time. Previous studies provide mechanistic plausibility for this reciprocal relationship, showing that increased nocturnal awakenings and sleep fragmentation can impair emotion regulation through the hyperactivation of the amygdala [29]. Notably, this bidirectional effect was specific to irritability, likely because irritability is more directly coupled with the “arousal system” than internalized symptoms such as depression. Unlike cognitive-heavy symptoms, nocturnal awakenings trigger immediate amygdala hyperreactivity and a failure in top-down prefrontal control [30], manifesting as acute reactive irritability. This heightened physiological arousal further disrupts sleep maintenance, reinforcing a self-perpetuating reciprocal loop [31]. Clinically, these findings suggest that the relationship between behavioral and psychological symptoms of dementia and sleep can be characterized as a vicious circle, where nocturnal awakenings serve as an early warning signal of worsening irritability.

Consistent with this interpretation, previous studies in patients with Alzheimer disease have shown that on nights when patients awakened their caregivers, they exhibited more behavioral and psychological symptoms of dementia the following morning. Additionally, patients who had difficulty falling asleep displayed more behavioral and psychological symptoms of dementia at night [27]. Similarly, Kuzmik et al [12] demonstrated that higher behavioral and psychological symptoms of dementia were associated with sleep disturbances, especially prolonged sleep latency. Thus, the relationship between the behavioral and psychological symptoms of dementia and sleep patterns seems to be complex and mutually reinforcing, necessitating the integration of sleep management into dementia symptom care programs.

The behavioral and psychological symptoms of dementia represent a primary burden for caregivers of older adults with dementia [7], and sleep disturbances affect the quality of life of both the primary caregiver and the older adult with dementia, contributing to cognitive decline and difficulties with community-based care [32]. Therefore, interventions to decrease these symptoms should be initiated early in the course of the symptoms, and interventions to improve sleep quality should be promoted simultaneously. Pharmacological interventions to address the behavioral and psychological symptoms of dementia are less accessible owing to the prescription process and may have side effects; therefore, nonpharmacologic interventions are recommended, such as reminiscence and music therapy, as they can be easily implemented by caregivers [33,34]. In particular, recent reports suggest that nonpharmacological interventions are more effective when customized to reflect the characteristics and preferences of older adults with dementia, compared with standardized interventions that are applied uniformly across all [35]. This highlights the need to develop and test nonpharmacological interventions aimed at improving sleep quality and alleviating behavioral and psychological symptoms of dementia in older adults with dementia.

This study has important implications for the field. To the best of our knowledge, this is the first study to explore the relationship between the behavioral and psychological symptoms of dementia and sleep patterns through panel vector autoregressive analysis. Sleep patterns were also observed using actigraphy technology, not subjective surveys.

### Limitations

This study has some limitations. First, behavioral and psychological symptoms of dementia were assessed using caregiver-recorded daily diaries, which may be vulnerable to recall bias owing to retrospective reporting. Future studies may consider ecological momentary assessment approaches using mobile apps to enable real-time symptom recording and reduce recall bias. Second, data on medications used by older adults to manage behavioral and psychological symptoms of dementia and sleep, as well as symptom-driven medication changes over time, were not available. Although the panel vector autoregressive model addresses time-invariant individual heterogeneity by modeling within-person temporal dynamics, it cannot fully adjust for unmeasured time-varying confounders, such as changes in medication use or comorbidity severity over time. Consequently, such unobserved temporal changes may have influenced both behavioral and psychological symptoms of dementia and sleep patterns and should be considered when interpreting the findings.

Third, the relatively short 14-day observation period limited the evaluation of longer lag structures and seasonal effects on sleep patterns. Future studies using longer-term longitudinal data are needed to examine extended lag dynamics and potential seasonal influences. Furthermore, the lack of observed bidirectionality in behavioral and psychological subsymptoms of dementia, other than irritability, may be due to their longer-term trajectories, which might necessitate more extended observation and larger cohorts to detect subtle daily fluctuations. Some statistically significant associations were characterized by small effect sizes, likely reflecting the chronic and progressive nature of dementia, in which abrupt day-to-day changes in behavioral and psychological symptoms of dementia and sleep are uncommon. In this context, the short observation period may have captured only subtle temporal fluctuations, and future studies with longer follow-up periods are needed to examine whether such small changes accumulate over time and become clinically meaningful.

Finally, this study relied on convenience sampling and included a relatively small sample, which may limit the generalizability of the findings. Moreover, although the overall participation rate (154/175, 88%) was relatively high for studies involving older adults with dementia, participant attrition occurred during the study period owing to reasons such as refusal to continue using the actigraphy device or hospitalization. Given that these reasons for dropout may have been more common among participants with worsening symptoms, the final analytic sample may underrepresent individuals with more severe conditions. This potential selection bias, combined with the modest sample size and wide 95% CIs for several estimates, may have reduced statistical power and increased the risk of both type I and type II errors.

### Conclusions

By integrating a linear mixed model and a panel vector autoregressive model, this study provides comprehensive evidence that accounts for individual variability and examines the bidirectional relationships between behavioral and psychological symptoms of dementia and sleep disturbances. This approach enhances the robustness of our Granger causal inferences. Panel vector autoregressive analysis demonstrated that the variables had an intricate and bidirectional relationship. This suggests that addressing sleep disturbances may mitigate certain behavioral and psychological symptoms of dementia, and vice versa.

The findings imply the importance of holistic approaches to dementia care management. One potential pathway to mitigate symptoms involves detecting behavioral and psychological symptoms of dementia onset and implementing timely interventions to improve both sleep and these symptoms of dementia. Given that these findings highlight the impact of sleep patterns on behavioral and psychological symptoms of dementia, there is a critical need for interventions aimed at improving sleep quality in older adults with dementia. However, the availability of effective nonpharmacologic interventions to enhance sleep quality in older adults with dementia remains limited, highlighting the need for future research to develop and validate such interventions.

## Appendix

Multimedia Appendix 1 Supplementary tables contain the lag order of the panel vector autoregression models, participants’ baseline characteristics, and linear mixed model results examining the relationships between sleep patterns and behavioral and psychological symptoms of dementia.

## Abbreviations

ICC: intraclass correlation coefficient
