# Supplementary material for: Dynamic Relationship Between Sleep Patterns and Behavioral and Psychological Symptoms of Dementia: Longitudinal Observational Study
Source: JMIR Aging. 2026 Mar 23;9:e80422. doi: 10.2196/80422 (PMC13054222; doi:10.2196/80422)
Supplement: Multimedia Appendix 1 [file aging_v9i1e80422_app1.docx]

**Supplementary table legend**

**Table S1.** Lag order of the panel vector autoregressive models

**Table S2.** Participants’ general characteristics at baseline

**Table S3.** Linear mixed model estimation results for total behavioral and psychological symptoms of dementia by sleep pattern

**Table S4**. Linear mixed model estimation results for sleep patterns by total behavioral and psychological symptoms of dementia

**Table S5**. Linear mixed model estimation results for the behavioral and psychological sub-symptoms of dementia by sleep patterns

**Table S6**. Linear mixed model estimation results for sleep patterns by the behavioral and psychological sub-symptoms of dementia

**Table S1.** Lag order of the panel vector autoregressive models

| **Lag** | **CD** | **J** | **J *p*-value** | **MBIC** | **MAIC** | **MQIC** |
| --- | --- | --- | --- | --- | --- | --- |
| 1 | 0.998 | 222.629 | 0.676 | -1327.789 | -243.371 | -660.553 |
| 2 | 0.990 | 126.932 | 0.720 | -784.687 | -147.068 | -392.364 |
| 3 | 0.744 | 37.871 | 0.962 | -328.108 | -72.129 | -170.606 |
| 4 | 0.218 | 18.408 | 0.495 | -108.021 | -19.592 | -53.612 |

Abbreviations: CD, correlated disturbance; MBIC, modified Bayesian Information Criterion; MAIC, modified Akaike Information Criterion; MQIC, modified Hannan–Quinn Information Criterion

**Table S2.** Participants’ general characteristics at baseline

| **Variables** | **Value (N=154)**  **N (%) or Mean (SD)** |
| --- | --- |
| Age (years), mean (SD) | 81.34 (5.97) |
| Sex, n (%) |  |
| Men | 61 (39.6) |
| Women | 93 (60.4) |
| Marital status, n (%) |  |
| Married | 90 (58.4) |
| Bereaved or divorced | 64 (41.6) |
| Education level, n (%) |  |
| Elementary school or below | 76 (49.4) |
| Middle school | 15 (9.7) |
| High school | 37 (24.0) |
| College or above | 26 (16.9) |
| ADL, mean (SD) | 10.68 (3.87) |
| MMSE, mean (SD) | 16.86 (5.84) |
| Sedative (yes), n (%) | 56 (38.3) |
| Dementia type, n (%) |  |
| Alzheimer’s disease | 76 (49.4) |
| Lewy body dementia | 60 (39.0) |
| Vascular dementia | 24 (15.6) |
| Other dementia | 34 (22.1) |

Abbreviations: ADL, activities of daily living; MMSE, Mini-Mental State Examination; SD, standard deviation.

**Table S3.** Linear mixed model estimation results for total behavioral and psychological symptoms of dementia by sleep pattern

|  | **Dependent variables** | | | | |
| --- | --- | --- | --- | --- | --- |
| **Independent variables** | **Total behavioral and psychological symptoms of dementia** | | | | |
|  | ***β*** | ***p*** | **Random effect** ^a^ | | |
|  |  |  | **Intercept** | **Slope** | **ICC** |
| TST | -0.00002 | 0.881 | 0.586 | 0.027 | 0.497 |
| WASO | 0.00034 | 0.713 | 0.584 | 0.270 | 0.496 |
| NoA | 0.00268 | 0.304 | 0.584 | 0.272 | 0.497 |
| MAL | -0.02010 | 0.296 | 0.589 | 0.270 | 0.498 |

Abbreviations: MAL, mean awakening length; NoA, number of awakenings; TST, total sleep time; WASO, wake after sleep onset.

^a^ The random effect intercept reflects differences in symptoms between individuals, while the random slope shows that the effect of previous day's symptoms varies for each individual. The random slope was statistically significant.

**Table S4**. Linear mixed-model estimation results for sleep patterns by total behavioral and psychological symptoms of dementia

|  | **Dependent variables** | | | | | | | | | | | | | | | | | | |  |
| --- | --- | --- | --- | --- | --- | --- | --- | --- | --- | --- | --- | --- | --- | --- | --- | --- | --- | --- | --- | --- |
|  | **TST** | | | | | **WASO** | | | | | **NoA** | | | | | **MAL** | | | | |
| **Independent variables** | ***β*** | ***p*** | **Random effect** | | | ***β*** | ***p*** | **Random effect** | | | ***β*** | ***p*** | **Random effect** | | | ***β*** | ***p*** | **Random effect** | | |
|  |  |  | **Intercept** | **Slope** | **ICC** |  |  | **Intercept** | **Slope** | **ICC** |  |  | **Intercept** | **Slope** | **ICC** |  |  | **Intercept** | **Slope** | **ICC** |
| Total behavioral and psychological  symptoms of dementia | 8.28780 | 0.110 | 15049.080 | 0.0195 | 0.292 | 0.38795 | 0.490 | 218.404 | 0.0213 | 0.339 | 0.20045 | 0.330 | 34.847 | 0.018 | 0.391 | 0.02316 | 0.295 | 0.107 | 0.005 | 0.097 |

Abbreviations: MAL, mean awakening length; NoA, number of awakenings; TST, total sleep time; WASO, wake after sleep onset.

^a^ The random effect intercept reflects differences in symptoms between individuals, while the random slope shows that the effect of the previous day's symptoms varies for each individual. The random slope was statistically significant.

**Table S5a**. Linear mixed-model estimation results for the behavioral and psychological subsymptoms of dementia by sleep patterns

|  | **Dependent variables** | | | | | | | | | | | | | | | | | | | |
| --- | --- | --- | --- | --- | --- | --- | --- | --- | --- | --- | --- | --- | --- | --- | --- | --- | --- | --- | --- | --- |
| **Independent variables** | **Delusion** | | | | | **Hallucination** | | | | | **Agitation or aggression** | | | | | **Depression** | | | | |
|  | ***β*** | ***p*** | **Random effect** ^a^ | | | ***β*** | ***p*** | **Random effect** ^a^ | | | ***β*** | ***p*** | **Random effect** ^a^ | | | ***β*** | ***p*** | **Random effect** ^a^ | | |
|  |  |  | **Intercept** | **Slope** | **ICC** |  |  | **Intercept** | **Slope** | **ICC** |  |  | **Intercept** | **Slope** | **ICC** |  |  | **Intercept** | **Slope** | **ICC** |
| TST | 0.00001 | 0.680 | 0.010 | 0.087 | 0.699 | -2.49E-06 | 0.901 | 0.006 | 0.128 | 0.835 | 0.00002 | 0.357 | 0.010 | 0.091 | 0.690 | 6.79E-08 | 0.998 | 0.019 | 0.090 | 0.692 |
| WASO | 0.00001 | 0.957 | 0.010 | 0.087 | 0.629 | 0.00018 | 0.326 | 0.006 | 0.128 | 0.797 | -0.00007 | 0.750 | 0.010 | 0.091 | 0.630 | 0.00017 | 0.508 | 0.019 | 0.090 | 0.570 |
| NoA | -0.00011 | 0.856 | 0.010 | 0.087 | 0.700 | 0.00061 | 0.218 | 0.006 | 0.129 | 0.836 | -0.00015 | 0.809 | 0.010 | 0.091 | 0.700 | 0.00050 | 0.472 | 0.019 | 0.090 | 0.691 |
| MAL | -0.00437 | 0.370 | 0.010 | 0.087 | 0.700 | 0.00064 | 0.870 | 0.006 | 0.128 | 0.835 | -0.00364 | 0.463 | 0.010 | 0.091 | 0.700 | 0.00367 | 0.491 | 0.019 | 0.090 | 0.691 |

**Table S5b**. Linear mixed-model estimation results for the behavioral and psychological subsymptoms of dementia by sleep patterns

|  | **Dependent variables** | | | | | | | | | | | | | | | | | | | |
| --- | --- | --- | --- | --- | --- | --- | --- | --- | --- | --- | --- | --- | --- | --- | --- | --- | --- | --- | --- | --- |
| **Independent variables** | **Anxiety** | | | | | **Euphoria or elation** | | | | | **Apathy** | | | | | **Disinhibition** | | | | |
|  | ***β*** | ***p*** | **Random effect** ^a^ | | | ***β*** | ***p*** | **Random effect** ^a^ | | | ***β*** | ***p*** | **Random effect** ^a^ | | | ***β*** | ***p*** | **Random effect** ^a^ | | |
|  |  |  | **Intercept** | **Slope** | **ICC** |  |  | **Intercept** | **Slope** | **ICC** |  |  | **Intercept** | **Slope** | **ICC** |  |  | **Intercept** | **Slope** | **ICC** |
| TST | 0.00003 | 0.224 | 0.015 | 0.104 | 0.725 | -0.00003 | 0.209 | 0.003 | 0.081 | 0.706 | 0.00006 | 0.031 | 0.024 | 0.111 | 0.720 | -0.00001 | 0.390 | 0.002 | 0.054 | 0.790 |
| WASO | 0.00016 | 0.491 | 0.015 | 0.104 | 0.633 | -0.00004 | 0.835 | 0.003 | 0.081 | 0.677 | -0.00009 | 0.746 | 0.024 | 0.111 | 0.593 | -0.00005 | 0.662 | 0.002 | 0.054 | 0.810 |
| NoA | 0.00040 | 0.545 | 0.015 | 0.104 | 0.725 | 8.10E-05 | 0.875 | 0.003 | 0.081 | 0.704 | 0.00070 | 0.339 | 0.024 | 0.111 | 0.722 | -0.00012 | 0.694 | 0.002 | 0.054 | 0.837 |
| MAL | 0.00620 | 0.228 | 0.015 | 0.103 | 0.724 | -0.00062 | 0.887 | 0.003 | 0.081 | 0.704 | -0.01348 | 0.015 | 0.024 | 0.111 | 0.725 | -0.00129 | 0.601 | 0.002 | 0.055 | 0.838 |

**Table S5c**. Linear mixed-model estimation results for the behavioral and psychological subsymptoms of dementia by sleep patterns

|  | **Dependent variables** | | | | | | | | | | | | | | | | | | | |
| --- | --- | --- | --- | --- | --- | --- | --- | --- | --- | --- | --- | --- | --- | --- | --- | --- | --- | --- | --- | --- |
| **Independent variables** | **Irritability** | | | | | **Aberrant motor behavior** | | | | | **Sleep and nighttime behavior** | | | | | **Appetite or eating disorders** | | | | |
|  | ***β*** | ***p*** | **Random effect** ^a^ | | | ***β*** | ***p*** | **Random effect** ^a^ | | | ***β*** | ***p*** | **Random effect** ^a^ | | | ***β*** | ***p*** | **Random effect** ^a^ | | |
|  |  |  | **Intercept** | **Slope** | **ICC** |  |  | **Intercept** | **Slope** | **ICC** |  |  | **Intercept** | **Slope** | **ICC** |  |  | **Intercept** | **Slope** | **ICC** |
| TST | 0.00007 | 0.016 | 0.014 | 0.109 | 0.707 | 0.00001 | 0.506 | 0.005 | 0.060 | 0.785 | -0.00004 | 0.123 | 0.012 | 0.110 | 0.690 | -0.00002 | 0.406 | 0.016 | 0.081 | 0.718 |
| WASO | 0.00002 | 0.945 | 0.015 | 0.108 | 0.621 | 0.00006 | 0.661 | 0.005 | 0.060 | 0.725 | 0.00001 | 0.969 | 0.012 | 0.111 | 0.624 | 0.00007 | 0.755 | 0.016 | 0.080 | 0.602 |
| NoA | 0.00046 | 0.512 | 0.015 | 0.108 | 0.707 | 0.00025 | 0.541 | 0.005 | 0.060 | 0.785 | -9.61E-05 | 0.892 | 0.012 | 0.111 | 0.692 | 0.00048 | 0.430 | 0.016 | 0.080 | 0.719 |
| MAL | -0.01124 | 0.039 | 0.015 | 0.108 | 0.706 | 0.00223 | 0.485 | 0.005 | 0.060 | 0.785 | 0.00142 | 0.801 | 0.012 | 0.111 | 0.691 | -0.00299 | 0.522 | 0.016 | 0.080 | 0.719 |

Abbreviations: MAL, mean awakening length; NoA, number of awakenings; TST, total sleep time; WASO, wake after sleep onset.

^a^ The random-effect intercept reflects differences in symptoms between individuals, while the random slope shows that the effect of the previous day's symptoms varies for each individual. The random slope was statistically significant.

**Table S6**. Linear mixed-model estimation results for sleep patterns by behavioral and psychological subsymptoms of dementia

|  | **Dependent variables** | | | | | | | | | | | | | | | | | | | |
| --- | --- | --- | --- | --- | --- | --- | --- | --- | --- | --- | --- | --- | --- | --- | --- | --- | --- | --- | --- | --- |
|  | **TST** | | | | | **WASO** | | | | | **NoA** | | | | | **MAL** | | | | |
| **Independent variables** | ***β*** | ***p*** | **Random effect** ^a^ | | | ***β*** | ***p*** | **Random effect** ^a^ | | | ***β*** | ***p*** | **Random effect** ^a^ | | | ***β*** | ***p*** | **Random effect** ^a^ | | |
|  |  |  | **Intercept** | **Slope** | **ICC** |  |  | **Intercept** | **Slope** | **ICC** |  |  | **Intercept** | **Slope** | **ICC** |  |  | **Intercept** | **Slope** | **ICC** |
| Delusion | 52.80208 | 0.013 | 15044.560 | 0.020 | 0.293 | 1.58586 | 0.493 | 218.592 | 0.021 | 0.339 | 0.97274 | 0.244 | 34.856 | 0.019 | 0.391 | 0.12920 | 0.203 | 0.111 | 0.006 | 0.104 |
| Hallucination | 38.50282 | 0.142 | 14995.910 | 0.020 | 0.291 | 2.12049 | 0.459 | 217.126 | 0.021 | 0.337 | 0.10062 | 0.922 | 34.579 | 0.019 | 0.389 | 0.17460 | 0.157 | 0.108 | 0.006 | 0.101 |
| Agitation or aggression | 28.85126 | 0.182 | 15434.370 | 0.019 | 0.298 | 3.60065 | 0.125 | 219.468 | 0.022 | 0.340 | 1.22234 | 0.149 | 34.835 | 0.019 | 0.391 | 0.02110 | 0.836 | 0.107 | 0.006 | 0.100 |
| Depression | 22.15717 | 0.271 | 15099.060 | 0.020 | 0.293 | -0.58724 | 0.788 | 217.417 | 0.021 | 0.337 | 0.07527 | 0.924 | 34.578 | 0.019 | 0.389 | 0.08480 | 0.378 | 0.106 | 0.006 | 0.099 |
| Anxiety | -7.35061 | 0.718 | 15370.820 | 0.020 | 0.296 | -0.55383 | 0.803 | 216.937 | 0.021 | 0.337 | -0.25718 | 0.748 | 34.458 | 0.019 | 0.388 | 0.11170 | 0.242 | 0.106 | 0.006 | 0.099 |
| Euphoria or elation | 3.14231 | 0.895 | 15379.040 | 0.020 | 0.297 | -1.23104 | 0.634 | 217.600 | 0.021 | 0.338 | -0.12368 | 0.894 | 34.587 | 0.019 | 0.389 | -0.04910 | 0.677 | 0.108 | 0.006 | 0.101 |
| Apathy | 12.50195 | 0.502 | 15115.330 | 0.020 | 0.293 | 3.68780 | 0.067 | 216.647 | 0.021 | 0.337 | 0.94460 | 0.193 | 34.547 | 0.018 | 0.389 | 0.15620 | 0.071 | 0.108 | 0.006 | 0.101 |
| Disinhibition | -73.25630 | 0.083 | 15321.510 | 0.020 | 0.296 | -0.76751 | 0.868 | 217.775 | 0.021 | 0.338 | -0.91004 | 0.582 | 34.749 | 0.019 | 0.390 | 0.08190 | 0.690 | 0.107 | 0.006 | 0.100 |
| Irritability | -3.59222 | 0.852 | 15430.170 | 0.020 | 0.297 | -0.68397 | 0.743 | 216.944 | 0.021 | 0.337 | 0.76883 | 0.306 | 34.836 | 0.019 | 0.391 | -0.12250 | 0.186 | 0.106 | 0.006 | 0.100 |
| Aberrant motor behavior | 55.94577 | 0.098 | 15308.310 | 0.019 | 0.296 | 2.90464 | 0.422 | 216.727 | 0.021 | 0.337 | 1.60642 | 0.220 | 34.604 | 0.019 | 0.390 | 0.04160 | 0.794 | 0.107 | 0.006 | 0.100 |
| Sleep and nighttime behavior | 9.55227 | 0.617 | 15486.870 | 0.020 | 0.298 | -0.61462 | 0.769 | 217.208 | 0.021 | 0.337 | -0.45156 | 0.548 | 34.548 | 0.019 | 0.389 | 0.00340 | 0.970 | 0.107 | 0.006 | 0.100 |
| Appetite or eating disorders | -16.38760 | 0.489 | 15399.670 | 0.020 | 0.297 | -2.29574 | 0.366 | 217.407 | 0.021 | 0.337 | -0.83290 | 0.364 | 34.608 | 0.019 | 0.389 | -0.08780 | 0.430 | 0.109 | 0.006 | 0.102 |

Abbreviations: MAL, mean awakening length; NoA, number of awakenings; TST, total sleep time; WASO, wake after sleep onset.

^a^ The random-effect intercept reflects differences in symptoms between individuals, while the random slope shows that the effect of the previous day's symptoms varies for each individual. The random slope was statistically significant.
